# Rationale and design of the OPTIMAL‐REPERFUSION trial: A prospective randomized multi‐center clinical trial comparing different fibrinolysis‐transfer percutaneous coronary intervention strategies in acute ST‐segment elevation myocardial infarction

**DOI:** 10.1002/clc.23582

**Published:** 2021-02-25

**Authors:** Zhongxiu Chen, Duolao Wang, Min Ma, Chen Li, Zhi Wan, Li Zhang, Ye Zhu, Mian Wang, Hua Wang, Sen He, Yong Peng, Jiafu Wei, Baotao Huang, Yong He, for the OPTIMAL‐REPERFUSION trial investigator

**Affiliations:** ^1^ Department of Cardiology West China Hospital of Sichuan University Chengdu China; ^2^ Department of Clinical Sciences Liverpool School of Tropical Medicine Liverpool UK; ^3^ Department of Emergency Medicine West China Hospital of Sichuan University Chengdu China

**Keywords:** pharmacoinvasive strategy, primary percutaneous coronary intervention, reduced‐dose fibrinolysis, ST‐elevation myocardial infarction

## Abstract

Primary percutaneous coronary intervention (PPCI), the preferred reperfusion strategy for all acute ST‐segment elevation myocardial infarction (STEMI) patients, is not universally available in clinical practice. Pharmacoinvasive strategy has been proposed as a therapeutic option in patients with STEMI when timely PPCI is not feasible. However, pharmacoinvasive strategy has potential delay between clinical patency and complete myocardial perfusion. The optimal reperfusion strategy for STEMI patients with anticipated PPCI delay according to current practice is uncertain. OPTIMAL‐REPERFUSION is an investigator‐initiated, prospective, multicenter, randomized, open‐label, superiority trial with blinded evaluation of outcomes. A total of 632 STEMI patients presenting within 6 hours after symptom onset and with an expected time of first medical contact to percutaneous coronary intervention (PCI) ≥120 minute will be randomized to a reduced‐dose facilitated PCI strategy (reduced‐dose fibrinolysis combined with simultaneous transfer for immediate invasive therapy with a time interval between fibrinolysis to PCI < 3 hours) or to standard pharmacoinvasive treatment. The primary endpoint is the composite of death, reinfarction, refractory ischemia, congestive heart failure, or cardiogenic shock at 30‐days. Enrollment of the first patient is planned in March 2021. The recruitment is anticipated to last for 12 to 18 months and to complete in September 2023 with 1 year follow‐up. The OPTIMAL‐REPERFUSION trial will help determine whether reduced‐dose facilitated PCI strategy improves clinical outcomes in patients with STEMI and anticipated PPCI delay. This study is registered with the ClinicalTrials.gov (NCT04752345).

## 1 INTRODUCTION

Early and successful restoration of myocardial perfusion after a ST‐elevation myocardial infarction (STEMI) is the most effective way to reduce the final infarct size and improve clinical outcomes. It is generally well‐accepted that primary percutaneous coronary intervention (PPCI) is the preferred reperfusion strategy for all STEMI patients when it can be performed within the guideline‐recommended time frame at PPCI‐capable centers.^1^, ^2^ However, PPCI is not universally available, and delay in performing percutaneous coronary intervention (PCI) is common in clinical practice, especially in low‐ and middle‐income countries.^3^, ^4^, ^5^ Pharmacoinvasive strategy, fibrinolysis combined with rescue PCI (in case of failed fibrinolysis) or routine early (3–24 hours) invasive strategy (in case of successful fibrinolysis), has been proposed as a therapeutic option for STEMI patients when timely PPCI is not feasible.^1^, ^2^ However, there is a potential time delay between clinical patency and complete myocardial perfusion in pharmacoinvasive strategy. Successful clinical reperfusion does not mean a good flow grade of thrombolysis in myocardial infarction (TIMI) and tissue perfusion. Studies have revealed that the proportion of TIMI flow grade < 3 confirmed by coronary angiography in patients with clinical patency was as high as 50%.^6^, ^7^, ^8^ Moreover, the re‐occlusion of the infarct‐related artery (IRA) and recurrent ischemia rate in clinical patency patients while waiting for PCI is high (nearly 30%).^9^ The optimal timing of coronary angiography after fibrinolysis is uncertain.^10^

Facilitated PCI strategy (fibrinolysis followed by immediate transfer for planned PCI within 90 to 120 minutes) can maximally reduce time delay, enable early treatment of patients with potential fibrinolysis failure and reinfarction of successful fibrinolysis, and thus theoretically achieve better outcomes. Previous studies demonstrated that facilitated PCI could significantly improve the clinical outcomes of STEMI patients compared with thrombolytic therapy.^11^, ^12^ Additionally, this strategy acquired significantly higher TIMI flow grade of IRA and better microcirculation perfusion compared with PPCI.^13^ However, increased bleeding risk, especially intracranial bleeding, became its Achilles' Heel and limited its clinical application. To date, no randomized clinical trial directly compared the regimen of facilitated PCI and pharmacoinvasive approach.^14^ More importantly, significant advances have occurred in pharmacological therapy and PCI technology in the past 20 years. The recent preferred use of radial‐artery access, bailout use of glycoprotein IIb/IIIa inhibitors (GPI) and introduction of newer fibrin‐specific thrombolytic agents are associated with fewer major bleeding complications. According to current practice, it is not clear whether reduced‐dose facilitated PCI strategy (reduced‐dose fibrinolysis combined with simultaneous transfer for immediate mechanical invasive therapy within 3 hours) is better than pharmacoinvasive approach or not. Randomized controlled trials are essentially needed to define the optimum reperfusion strategy in patients with STEMI and anticipated PPCI delay and help create evidence‐based practice on this controversial issue. The trial's hypothesis is that, in STEMI patients with anticipated PPCI delay, reduced‐dose facilitated PCI strategy is superior to pharmacoinvasive approach with respect to the clinical events over the duration of the trial (see Appendix S1).

## 2 METHODS

### 2.1 Study design

**FIGURE 1:**
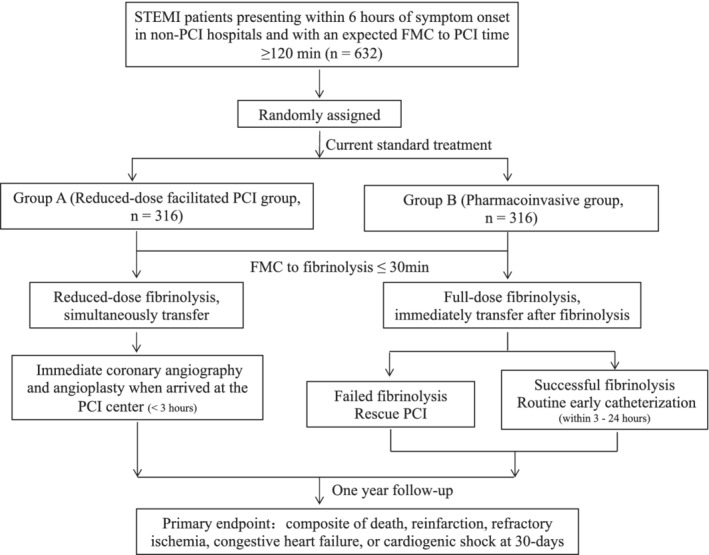
The flow chart of the study. FMC, first medical contact; PCI, percutaneous coronary intervention; STEMI, ST‐elevation myocardial infarction

OPTIMAL‐REPERFUSION is a prospective, multicenter, randomized, parallel group, open‐label, superiority trial to evaluate the efficacy and safety of reduced‐dose facilitated PCI strategy compared with pharmacoinvasive approach in patients with STEMI and anticipated PPCI delay in contemporary practices. The Executive Committee designed the protocol and is responsible for the conduct and oversight of the study. The trial is coordinated by the West China Hospital of Sichuan University, Chengdu, China. The trial plans to enroll around 632 patients at approximately 30 sub‐centers in the south‐western part of China. Participating centers and the principal investigators are listed in Table S1. The specific requirements of the sub‐centers are shown in the Appendix S1. Each selected sub‐center will compete for enrollment and each sub‐center will have a maximum enrollment of 60 cases. A flowchart depicting the trial design is shown in Figure 1. Given the apparent difference in time of transfer and invasive procedure between the two regimens, it will not be possible to blind the subjects and operators as to which strategy the patients will undergo. To minimize potential bias, a robust inclusion/exclusion criteria and clear endpoint definitions and boundaries will be used. Any deviation from the protocol in delivery of strategy will be carefully recorded, including any concomitant therapies. The protocol has been approved by institutional review boards in all participating centers. Written informed consent will be obtained from all participants (see Appendix S1).

### 2.2 Funding

The trial is funded by the Key Research and Development Programs of Sichuan Province (grant number: 2020YFS0244 and 2020YFS0242) and 1·3·5 project for disciplines of excellence–Clinical Research Incubation Project, West China Hospital, Sichuan University (grant number: 2021HXFH021), with additional support in the form of unrestricted, investigator‐initiated research grants from Tasly Pharmaceuticals. The authors are solely responsible for the design and conduct of this study, all study analyses, results interpretations, and drafting of the final report.

### 2.3 Patients

STEMI patients between 18 and 75 years of age who present within 6 hours after symptom onset to participating PCI‐incapable primary hospital and with an expected first medical contact (FMC) to PCI time ≥ 120 minute will be screened for study eligibility. Patients must have ≥2 mm ST‐segment elevation in 2 contiguous precordial leads, ≥1 mm ST‐segment elevation in 2 contiguous extremity leads, or new left bundle branch block with symptom onset persisting for more than 30 minute. Key exclusion criteria included any contraindication for fibrinolysis, cardiogenic shock before randomization, PCI within last 6 months, and previous coronary‐artery bypass surgery. A complete list of the exclusion criteria is provided in Table S2.

### 2.4 Randomization

Patients who satisfy the inclusion and exclusion criteria will be randomly assigned in a 1:1 fashion to either a ''reduced‐dose facilitated PCI group'' or ''pharmacoinvasive group''. Randomization is performed through an Interactive Web‐based Response System, which will be implemented to assign a randomization number to an eligible patient as well as to track enrollment across all centers, with a permuted block randomization scheme stratified by the time interval between disease onset and enrollment (less than 3 hours and 3–6 hours).

### 2.5 Treatment

All patients will receive an upfront loading dose of 300 mg aspirin and 300 mg clopidogrel in the emergency room of non‐PCI medical institutions. Potent adenosine diphosphate (ADP) receptor antagonists, prasugrel, and ticagrelor are not recommended. Patients who have already taken aspirin or ADP receptor antagonists ≤12 hours before screening will be given these agents on the following day. GPI use is not allowed before PCI. The bailout uses of GPI in the catheter lab or post‐catheterization is at the operator's discretion. Routine anticoagulation therapy after PCI is not recommended. Recombinant human prourokinase (rhPro‐UK, Tasly Pharmaceuticals, Shanghai, China), a urokinase precursor that represents a new generation of fibrin‐specific thrombolytic drugs with relatively few adverse reactions,^15^ is the thrombolytic agent used in both regimen groups. Comedication consists of unfractionated heparin given as 60 U/kg (up to a maximum of 4000 U) bolus before the thrombolytic agent and will be maintained at 12 U/kg/h (up to a maximum of 1000 U/h) until the catheterization. For coronary angiography, the radial‐artery approach is preferred. During the invasive procedure, an additional intravenous bolus of heparin could be administered if needed to obtain an activated clotting time of 200 to 250 seconds. Beta‐blockers, angiotensin‐converting enzyme inhibitors/angiotensin II receptor blockers, statins, and post‐interventional antiplatelet therapy will be administered to patients as outlined in the guidelines for myocardial infarction.^2^

### 2.6 Pharmacoinvasive group

Enrolled patients will be injected with 20 mg rhPro‐UK intravenously followed by intravenous infusion of 30 mg rhPro‐UK within 30 minute in the emergency department of non‐interventional hospitals. Transfer to a PCI‐capable center following fibrinolysis is indicated for all patients immediately after fibrinolysis. In case of insufficient ST resolution (less than 50% reduction in ST‐segment elevation) at 60–90 minute or clinically indicated by the presence of hemodynamic or electrical instability, or worsening ischemia, rescue PCI will be taken. Early routine catheterization within 3–24 hours for successful fibrinolysis will be performed. PCI will be performed when persistent occlusion or substantial stenosis of the IRA (either stenosis of ≥70% of the diameter of the artery or stenosis of 50% to 70% with thrombus, ulceration, or spontaneous dissection) is present. Direct stent implantation and new generation of drug‐eluting stents are recommended, and routine post‐dilation is not encouraged^16^, ^17^, ^18^ unless there is an obvious stent under‐expansion.

### 2.7 Reduced‐dose facilitated PCI group

Patients randomly assigned to the ''reduced‐dose facilitated PCI group'' will immediately start reduced‐dose thrombolysis treatment (intravenous injection of 20 mg rhPro‐UK followed by intravenous infusion of 10 mg rhPro‐UK within 30 minute) with simultaneous transfer to a PCI center through an affiliated ambulance of the participating local hospital. En route, participating non‐PCI center and PCI center will be contacted to confirm the potential feasibility of transfer and immediate PCI upon arrival. When arriving at the PCI centers, bypassing the emergency department will be strongly recommended and the patient will be brought straight to the catheterization laboratory, and immediate coronary angiography and angioplasty will be performed. The requirements of stent implantation are the same as the ''pharmacoinvasive group''.

### 2.8 Primary endpoint

The primary endpoint of the trial is the composite of death, reinfarction, refractory ischemia, congestive heart failure, and cardiogenic shock at 30‐days. Definitions of the end points are provided in Table 1.

**TABLE 1:** Endpoint definitions

Endpoint	Definition
Death	Death will be classified as cardiovascular or non‐cardiovascular. All deaths with a clear cardiovascular or unknown cause, will be classified as cardiac. However, within cardiac deaths, hemorrhagic deaths will be clearly identified. Only deaths due to a documented non‐cardiac cause (e.g., cancer) will be classified as non‐cardiac.
Reinfarction	Recurrent symptoms or signs of cardiac ischemia lasting more than 30 min with new ST‐T segment changes or Q‐wave in at least 2 contiguous leads or new onset LBBB and recurrent significant increase in cardiac enzyme levels. The increase in CK‐MB level is considered significant when it occurs after at least a ≥ 25% decrease in CK‐MB from a prior peak level and is >2 times the upper limit of normal (ULN) in the absence of coronary interventions, or > 5 times above the ULN after PCI.
Refractory ischemia	Symptoms of ischemia with ST‐deviation or definite T‐wave inversion persisting for at least 10 min despite enough antianginal drug occurring more than 12 hr after randomization.
Congestive heart failure	New or worsening congestive heart failure will be considered as patients presenting with at least one of the following conditions and requiring treatment with diuretics: 1) Pulmonary oedema/congestion on chest X‐ray without suspicion of a non‐cardiac cause; 2) Rales >1/3 up from the lung base; 3) Pulmonary capillary wedge pressure (PCWP) >25 mmHg; 4) Dyspnea with PO_2_ < 80 mmHg or O_2_ sat < 90% (no supplemental O_2_) in the absence of known lung disease.
Cardiogenic shock	The manifestation of vascular collapse and shock (systolic BP < 90 mmHg for at least 30 min or systolic BP > 90 mmHg after inotropic or intra‐aortic balloon support with a cardiac index <2.2 L/min/m^2^ or < 2.5 L/min/m^2^ after inotropic or intra‐aortic balloon support, peripheral signs of hypoperfusion, and chest X‐ray with pulmonary edema.
Major ventricular arrhythmia	Ventricular arrhythmias, occurring more than 6 hr after randomization, persisting for at least 30 sec, and accompanying with unstable hemodynamics that required electrical cardioversion/defibrillation.
Ischemia stroke	Defined as the presence of a new focal neurologic deficit thought to be vascular in origin, with signs or symptoms lasting more than 24 hr. It is strongly recommended (but not required) that an imaging procedure, such as, a computerized tomography (CT) or magnetic resonance imaging (MRI) be performed.
TIMI flow grade	TIMI flow grade 0 (no perfusion) refers to the absence of any antegrade flow beyond a coronary occlusion; TIMI flow grade 1 (penetration without perfusion) is faint antegrade coronary flow beyond the occlusion, with incomplete filling of the distal coronary bed; TIMI flow grade 2 (partial reperfusion) is delayed or sluggish antegrade flow with complete filling of the distal territory; and TIMI flow grade 3 (complete perfusion) is normal flow which fills the distal coronary bed completely.
TMPG	TMPG0: Failure of dye to enter the microvasculature; TMPG1: Dye slowly enters but fails to exit the microvasculature; TMPG2: Delayed entry and exit of dye from the microvasculature; and TMPG3: Normal entry and exit of dye from the microvasculature.

Abbreviations: BP, blood pressure; CK‐MB, creatine kinase‐myocardial band; LBBB, left bundle branch block; PCI, percutaneous coronary intervention; TIMI, thrombolysis in myocardial infarction; TMPG, TIMI myocardial perfusion grade.

### 2.9 Secondary endpoints

Secondary endpoints include the individual components of the primary endpoint, major ventricular arrhythmia, ischemia stroke, stent thrombosis, target vessel revascularization at 30 days and 1 year, complete epicardial and myocardial reperfusion [TIMI 3 epicardial flow and TIMI 3 myocardial reperfusion and complete (≥70%) ST‐segment resolution of the initial sum of ST‐segment elevation] after PCI, infarct size (assessed indirectly by the peak level of creatine kinase‐myocardial band), and left ventricular function assessed by echocardiography on the day before discharge, at 30 days and 1 year. The stent thrombosis and target vessel revascularization are defined in accordance with the Academic Research Consortium (ARC) definitions.^19^ Definitions of the endpoints are provided in Table 1.

### 2.10 Safety endpoint

The primary safety endpoint is the incidence of intracranial hemorrhage and major bleeding. All bleeding complications are classified using the Bleeding Academic Research Consortium (BARC) definition.^20^ Major bleeding is categorized as type 3 or 5 (type 3 indicating bleeding with a decrease in the hemoglobin of >3 g per deciliter, any transfusion, cardiac tamponade, or intracranial or ocular involvement; and type 5 indicating fatal bleeding). All bleeding needs to describe the bleeding site.

### 2.11 Ancillary studies

Additional assessment includes economic analysis and health‐related quality of life. Economic analysis aims to estimate the cost consequences and the cost‐effectiveness of reduced‐dose facilitated PCI strategy compared to current standard care. The total costs during the first 12 months include resources used during the first hospitalization including transportation and catheterization procedures, medications, examinations, management of complications and subsequent hospital admissions for cardiovascular problems in the first year after STEMI will be collected. The patients will register their use of health resources between each follow‐up. Health‐related quality of life will be collected at baseline and at each follow‐up visit and will be measured with EQ‐5D questionnaire.

### 2.12 Follow‐up

All study participants will be followed up at 30 ± 5 days and 1 year ±30 days after enrolment. Telephone follow‐up will be obtained at 14 ± 3 days and 90 ± 14 days after enrolment. At 180 ± 30 days, a telephone or site follow‐up visit will be carried out. The data collection and monitoring are shown in the Appendix S1. Clinical endpoints will be adjudicated by a clinical events committee blinded to treatment group assignment.

### 2.13 Sample size

The sample size is estimated based on the primary study endpoint. The primary endpoint event definitions and rate for the ''pharmacoinvasive group'' was estimated according to WEST (Which Early STEMI Therapy) study,^21^ in which, the 30‐day composite primary event (death 1%, reinfarction 5.8%, refractory ischemia 2.9%, congestive heart failure 14.4%, cardiogenic shock 3.8%) occurred in 24% of patients in the pharmacoinvasive group.

On the basis of the potential fibrinolysis failure rate is 30%–40%,^22^ and the early re‐occlusion and recurrent ischemia rate after initial successful fibrinolysis is as high as 30% in the standard therapy group,^9^ the expected relative risk reduction in the primary end point with ''reduced‐dose facilitated PCI group'' relative to the standard therapy group was estimated as 40% (14.4% for the event rate). With a power of 85% and a 2‐sided alpha of 0.05, the required sample size is 301 patients per group, for a total sample size of 601 patients. With an anticipated loss to follow‐up of approximately 5%, the target sample size was set at 632 patients. Moreover, sample size re‐estimation will be conducted when approximately 50% of participants have been recruited (Appendix S1).

### 2.14 Statistical analysis

Primary trial analyses will be intention to treat (ITT) and additional analyses will also be done on the per protocol population (PP). ITT population consists of all randomized subjects with valid informed consent. PP population is a subset of the ITT population in which subjects with major protocol deviations will be excluded. Protocol deviations will be defined in the statistical analysis plan.

A log binomial model (a generalized linear model [GLM]) will be used to analyze the primary endpoint. The GLM will include the treatment arm as the study variable, from which the relative risk (RR) of having a primary outcome between intervention and control together with 95% confidence interval (CI) will be derived. Covariate adjusted analysis of the primary endpoint will also be performed within the GLM framework with treatment arm as the study variable and the time to randomization, sex, weight, systolic blood pressure, infarct location, Killip class, and a history of diabetes or hypertension as covariates. Adjusted RR together with their 95% CI will be derived from the covariate adjusted GLM model. Subgroup analysis will also be performed for the above pre‐specified covariates.

The secondary binary and continuous outcomes will be analyzed similarly using GLM models. For GLM analysis of a continuous endpoint such as left ventricular function, normal distribution and identity link functions will be used; For GLM analysis of a binary outcome, (such as, having a primary endpoint), binomial distribution and log link functions will be used.

For the analysis of time‐to‐event outcome, the Kaplan–Meier curves will be presented and compared by the log rank test by treatment group, and hazard ratio and its 95% CI will be calculated using Cox regression model with the treatment arm as the study variable.

Generalized linear mixed model (GLMMIX) model will be employed to analyze the outcomes with repeated measurements. The model will have treatment, visit, interaction between treatment and visit as fixed effects, and subjects as random effect. For the analysis of binary secondary outcomes with repeated measurements, the GLMMIX model will have a binomial distribution and logit link function. The odds ratio between 2 treatment arms at each visit together with its 95% CI will be derived from the GLMMIX model. For the analysis of continuous secondary outcomes with repeated measurements, the GLMMIX model will have a normal distribution and identify link function. The mean difference between 2 treatment arms at each visit together with its 95% CI will be derived from the GLMMIX model.

Continuous variables will be summarized using number of observations, mean (standard deviation) or median (inter quartile range) as appropriate; categorical variables will be summarized by the number and percentage of events. Time‐to‐event variables will also be summarized by the number (%) of patients having an event and events per 100 person‐years by treatment arm. Analyses of the potential adverse effects of the test strategy will be done in the safety population.

All analyses will be described in detail in the statistical analysis plan. All statistical analyses will be performed using the SAS 9.4. The trial results will be reported following the Consolidated Standards of Reporting Trials (CONSORT) guidelines for randomized clinical trials.

### 2.15 Trial status

The trial is now actively preparing at West China Hospital of Sichuan University and other sub‐centers. The first patient will be planned to enroll in March 2021. The recruitment will last for 12 to 18 months and is planned to complete in September 2022.

## 3 DISCUSSION

In countries and areas, with large, sparse population and not well‐organized STEMI networks for PPCI, many patients with STEMI present to hospitals which do not have PCI facilities. Transfer for PPCI in a timely manner is associated with favorable clinical outcomes. However, the time for most patients who require transfer for PPCI far exceeds the guideline‐recommended 90‐minute time limit. Pharmacoinvasive strategy therefore remains a valuable therapeutic option in rural areas with long transportation delays in many institutions. The OPTIMAL‐REPERFUSION trial will compare the clinical efficacy and safety of reduced‐dose facilitated PCI strategy versus pharmacoinvasive strategy, and optimize treatment strategy for STEMI patients with anticipated PPCI delay according to current practice. The reduced‐dose facilitated PCI strategy, which combines reduced‐dose fibrinolysis with simultaneous transfer for immediate invasive therapy, can achieve shorter time delays between symptom onset and complete revascularization, and is expected to result in improved clinical outcomes compared with 3–24 hours regimen of pharmacoinvasive strategy.

### 3.1 Reduced‐dose fibrinolysis in STEMI patients

The STREAM trial (Strategic Reperfusion Early After Myocardial Infarction) showed that a pharmacoinvasive strategy could be a reasonable alternative to PPCI in STEMI patients presenting ≤3 hours of symptom onset and with an expected time delay from FMC to PPCI >1 hour. The only downside of the pharmacoinvasive arm was that its rate of intracranial hemorrhage with full‐dose Tenecteplase was 5 times higher than that of the PPCI group. However, the difference was not significant after a trial protocol amendment reducing Tenecteplase dose by 50% in the elderly.^23^ The ongoing STREAM‐2 study^24^ (NCT02777580) will further compare the efficacy and safety of pharmacoinvasive strategy with half‐dose Tenecteplase as compared to routine PPCI in STEMI patients ≥60 years presenting within 3 hours from symptom onset. Additionally, in an observational registry study in the United States in patients with STEMI with long PCI‐related delays, a pharmacoinvasive strategy utilizing half‐dose fibrinolysis (97% tenecteplase, 3% reteplase) combined with transfer for PCI achieved similar efficacy outcomes as PPCI without increased bleeding risk.^25^ More recently, EARLY‐MYO trial (Early Routine Catheterization After Alteplase Fibrinolysis Versus Primary PCI in Acute STEMI) demonstrated that a pharmacoinvasive strategy with half‐dose alteplase and timely PCI offered more complete epicardial and myocardial reperfusion when compared with PPCI for patients with STEMI at low risk presenting ≤6 hours after symptom onset and for whom the expected PCI‐related delay was ≥60 minutes and no significant differences in rates of major bleeding events or intracranial hemorrhage.^8^ Thus, reduced‐dose fibrinolytic regimen might be a safe and effective option for pharmacoinvasive treatment in eligible patients with STEMI.

### 3.2 Optimal timing of coronary angiography after fibrinolysis

A meta‐analysis of seven randomized controlled trials consisting of NORDISTEMI (NORwegian study on DIstrict treatment of ST‐elevation myocardial infarction),^7^ CARESS‐in‐AMI (combined Abciximab reteplase stent study in acute myocardial infarction) study,^26^ and the TRANSFER‐AMI (trial of routine angioplasty and stenting after fibrinolysis to enhance reperfusion in acute myocardial infarction),^6^ demonstrated that early transfer for catheterization after fibrinolysis was associated with a statistically significant reduction in the incidence of death and reinfarction at 30 days and at 1 year compared with either an ischemia‐driven catheterization or delayed catheterization at 24 hours to 2 weeks, most notably in higher‐risk patients.^27^ Guidelines for the management of STEMI recommended early routine angiography with subsequent PCI (if needed) after fibrinolysis.^1^, ^2^ However, the optimal timing of invasive assessment after fibrinolysis and the association with clinical outcomes is uncertain. A patient‐level data meta‐analysis of 6 randomized trials, with a median time to angiography <12 h after fibrinolysis, revealed that very early angiography (<2 h) after fibrinolysis was not associated with an increased risk of 30‐day death/reinfarction or in‐hospital major bleeding, and angiography within 4 h after fibrinolysis was associated with reduced 30‐day recurrent ischemia.^10^ In addition, this meta‐analysis showed that 30‐day and 1‐year death/reinfarction and 30‐day recurrent ischemia increased significantly with increasing symptom onset to angiography time.

Controversially, when compared with PPCI, ASSENT‐4 PCI (primary versus Tenecteplase‐facilitated PCI in patients with STEMI) and FINESSE (facilitated intervention with enhanced reperfusion speed to stop events) trial showed that facilitated PCI did not improve the endpoint events (death, congestive heart failure, cardiogenic shock, and recurrent myocardial infarction) of STEMI patients, and increase bleeding complications.^28^, ^29^ Due to the associated increased bleeding risk, very early catheterization after administration of fibrinolytic therapy is not routinely recommended unless for patients with evidence of failed fibrinolysis when rescue PCI would be appropriate. However, suboptimum antithrombotic co‐therapy during reperfusion (not given an infusion of heparin after a single intravenous bolus and not routinely given clopidogrel) in the test arm of ASSENT‐4 PCI trial, and additional time delay required for administration of lytic agent, planned use of GPI in all patients, and antiplatelet therapy with aspirin alone in FINESSE trial could in part explain the worse clinical outcome noted in these patients. In addition, most patients (45% in ASSENT‐4 PCI and 60% in FINESSE trial) were enrolled in PCI hospitals, which may lead to the observed results that cannot be generalizable to non‐PCI centers with long delays for transfer patients for PCI.

Since the publication of these trials, significant advances have occurred in pharmacological therapy and PCI technology. Therefore, in patients with STEMI presenting in non‐PCI centers and with anticipated PPCI delay, fibrinolysis followed by very early angiography (<2 to 3 hours) may be a feasible strategy according to current practice. Moreover, many studies demonstrated that approximately 50% of the successful fibrinolysis patients of standard pharmacoinvasive strategy did not achieve optimal perfusion (TIMI flow grade 3),^6^, ^7^, ^8^, ^23^, ^26^, ^30^ not to mention patients of failed fibrinolysis, and very early revascularization is of great clinical significance for these suboptimal perfusion patients, whom might potentially benefit from the reduced‐dose facilitated PCI strategy.

The antithrombotic regimen in OPTIMAL‐REPERFUSION includes both aspirin, clopidogrel, and heparin on top of rhPro‐UK. This antithrombotic regimen is more aggressive than the regimen used in the test arm of the ASSENT‐4 PCI trial, which noted more ischemic complications than PPCI group. Moreover, the reduced‐dose rhPro‐UK in the test arm of OPTIMAL‐REPERFUSION trial, combined with routine use of radial‐artery access and bailout use of GPI, aims to reduce bleeding risk.

In conclusion, the OPTIMAL‐REPERFUSION study is designed to provide important information on whether reduced‐dose facilitated PCI strategy is safe, cost‐effective, and superior to the standard pharmacoinvasive approach with 3 to 24 hours invasive regimen.

## CONFLICT OF INTEREST

The authors declare no potential conflict of interest.

## Supporting information


**Appendix** S1**: Supporting Information**

## Data Availability

The data underlying the findings of the paper are freely available on request through the authors themselves. Yong He (Department of Cardiology, West China Hospital of Sichuan University, 37 Guo Xue Xiang, Chengdu, Sichuan, 610041, China; e‐mail: heyong_huaxi@163.com) should be contacted to request the data.

## References

[1] O'Gara PT , Kushner FG , Ascheim DD , et al. 2013 ACCF/AHA guideline for the management of ST‐elevation myocardial infarction: a report of the American College of Cardiology Foundation/American Heart Association task force on practice guidelines. Circulation. 2013;127(4):e362‐e425. 23247304 10.1161/CIR.0b013e3182742cf6

[2] Ibanez B , James S , Agewall S , et al. 2017 ESC guidelines for the management of acute myocardial infarction in patients presenting with ST‐segment elevation: the task force for the management of acute myocardial infarction in patients presenting with ST‐segment elevation of the European Society of Cardiology (ESC). Eur Heart J. 2018;39(2):119‐177. 28886621 10.1093/eurheartj/ehx393

[3] Xun YW , Yang JG , Song L , et al. In‐hospital delay to primary angioplasty for patients with ST‐elevated myocardial infarction between cardiac specialized hospitals and non‐specialized hospitals in Beijing. China Chin Med J. 2010;123(7):800‐805. 20497667

[4] Kaifoszova Z , Kala P , Alexander T , et al. Stent for life initiative: leading example in building STEMI systems of care in emerging countries. EuroIntervention. 2014;10:87‐95. 10.4244/EIJV10STA14 25256540

[5] Mehta S , Granger C , Grines CL , et al. Confronting system barriers for ST‐ elevation MI in low and middle income countries with a focus on India. Indian Heart J. 2018;70(1):185‐190. 29455776 10.1016/j.ihj.2017.06.020 PMC5903067

[6] Cantor WJ , Fitchett D , Borgundvaag B , et al. Routine early angioplasty after fibrinolysis for acute myocardial infarction. N Engl J Med. 2009;360(26):2705‐2718. 19553646 10.1056/NEJMoa0808276

[7] Bohmer E , Hoffmann P , Abdelnoor M , et al. Efficacy and safety of immediate angioplasty versus ischemia‐guided management after thrombolysis in acute myocardial infarction in areas with very long transfer distances results of the NORDISTEMI (NORwegian study on DIstrict treatment of ST‐elevation myocardial infarction). J Am Coll Cardiol. 2010;55(2):102‐110. 19747792 10.1016/j.jacc.2009.08.007

[8] Pu J , Ding S , Ge H , Han Y , et al. Efficacy and safety of a Pharmaco‐invasive strategy with half‐dose Alteplase versus primary angioplasty in ST‐segment‐elevation myocardial infarction: EARLY‐MYO trial (early routine catheterization after Alteplase fibrinolysis versus primary PCI in acute ST‐segment‐elevation myocardial infarction). Circulation. 2017;136(16):1462‐1473. 28844990 10.1161/CIRCULATIONAHA.117.030582

[9] Pilote L , Miller DP , Califf RM . Recurrent ischemia after thrombolysis for acute myocardial infarction. Am Heart J. 2001;141(4):559‐565. 11275920 10.1067/mhj.2001.113226

[10] Madan M , Halvorsen S , Di Mario C , et al. Relationship between time to invasive assessment and clinical outcomes of patients undergoing an early invasive strategy after fibrinolysis for ST‐segment elevation myocardial infarction: a patient‐level analysis of the randomized early routine invasive clinical trials. JACC Cardiovasc Interv. 2015;8:166‐174. 25616922 10.1016/j.jcin.2014.09.005

[11] Thiele H , Engelmann L , Elsner K , et al. Comparison of pre‐hospital combination‐fibrinolysis plus conventional care with pre‐hospital combination‐fibrinolysis plus facilitated percutaneous coronary intervention in acute myocardial infarction. Eur Heart J. 2005;26(19):1956‐1963. 16061501 10.1093/eurheartj/ehi432

[12] Le May MR , Wells GA , Labinaz M , et al. Combined angioplasty and pharmacological intervention versus thrombolysis alone in acute myocardial infarction (CAPITAL AMI study). J Am Coll Cardiol. 2005;46(3):417‐424. 16053952 10.1016/j.jacc.2005.04.042

[13] Peters S , Truemmel M , Koehler B . Facilitated PCI by combination fibrinolysis or upstream tirofiban in acute ST‐segment elevation myocardial infarction: results of the Alteplase and Tirofiban in acute myocardial infarction (ATAMI) trial. Int J Cardiol. 2008;130(2):235‐240. 18055037 10.1016/j.ijcard.2007.08.048

[14] Fazel R , Joseph TI , Sankardas MA , et al. Comparison of reperfusion strategies for ST‐segment‐elevation myocardial infarction: a multivariate network meta‐analysis. J Am Heart Assoc. 2020;9(12):e015186. 32500800 10.1161/JAHA.119.015186 PMC7429064

[15] Zhao L , Zhao Z , Chen X , et al. Safety and efficacy of prourokinase injection in patients with ST‐elevation myocardial infarction: phase IV clinical trials of the prourokinase phase study. Heart Vessels. 2018;33(5):507‐512. 29209778 10.1007/s00380-017-1097-x

[16] Zhang ZJ , Marroquin OC , Stone RA , et al. Differential effects of post‐dilation after stent deployment in patients presenting with and without acute myocardial infarction. Am Heart J. 2010;160(5):979‐986. 21095289 10.1016/j.ahj.2010.07.007 PMC3003443

[17] Yamaji K , Brugaletta S , Sabate M , et al. Effect of post‐dilatation following primary PCI with Everolimus‐eluting Bioresorbable scaffold versus Everolimus‐eluting metallic stent implantation: an angiographic and optical coherence tomography TROFI II substudy. JACC Cardiovasc Interv. 2017;10(18):1867‐1877. 28935079 10.1016/j.jcin.2017.07.035

[18] Zhang ZJ , Ma L , Wan J , Kip KE . Stent post‐dilation for patients with ST‐elevation myocardial infarction undergoing primary percutaneous coronary intervention. Catheter Cardiovasc Interv. 2014;84(4):682‐683. 24259434 10.1002/ccd.25299

[19] Cutlip DE , Windecker S , Mehran R , et al. Clinical end points in coronary stent trials: a case for standardized definitions. Circulation. 2007;115(17):2344‐2351. 17470709 10.1161/CIRCULATIONAHA.106.685313

[20] Mehran R , Rao SV , Bhatt DL , et al. Standardized bleeding definitions for cardiovascular clinical trials: a consensus report from the bleeding academic research consortium. Circulation. 2011;123(23):2736‐2747. 21670242 10.1161/CIRCULATIONAHA.110.009449

[21] Armstrong PW . A comparison of pharmacologic therapy with/without timely coronary intervention vs. primary percutaneous intervention early after ST‐elevation myocardial infarction: the WEST (which early ST‐elevation myocardial infarction therapy) study. Eur Heart J. 2006;27(13):1530‐1538. 16757491 10.1093/eurheartj/ehl088

[22] Gershlick AH , Stephens‐Lloyd A , Hughes S , et al. Rescue angioplasty after failed thrombolytic therapy for acute myocardial infarction. N Engl J Med. 2005;353(26):2758‐2768. 16382062 10.1056/NEJMoa050849

[23] Armstrong PW , Gershlick AH , Goldstein P , et al. Fibrinolysis or primary PCI in ST‐segment elevation myocardial infarction. N Engl J Med. 2013;368(15):1379‐1387. 23473396 10.1056/NEJMoa1301092

[24] Armstrong PW , Bogaerts K , Welsh R , et al. The second strategic reperfusion early after myocardial infarction (STREAM‐2) study optimizing pharmacoinvasive reperfusion strategy in older ST‐elevation myocardial infarction patients. Am Heart J. 2020;226:140‐146. 32553932 10.1016/j.ahj.2020.04.029

[25] Larson DM , Duval S , Sharkey SW , et al. Safety and efficacy of a pharmaco‐invasive reperfusion strategy in rural ST‐elevation myocardial infarction patients with expected delays due to long‐distance transfers. Eur Heart J. 2012;33(10):1232‐1240. 22041553 10.1093/eurheartj/ehr403

[26] Di Mario C , Dudek D , Piscione F , et al. Immediate angioplasty versus standard therapy with rescue angioplasty after thrombolysis in the combined Abciximab REteplase stent study in acute myocardial infarction (CARESS‐in‐AMI): an open, prospective, randomised, multicentre trial. Lancet. 2008;371(9612):559‐568. 18280326 10.1016/S0140-6736(08)60268-8

[27] Borgia F , Goodman SG , Halvorsen S , et al. Early routine percutaneous coronary intervention after fibrinolysis vs. standard therapy in ST‐segment elevation myocardial infarction: a meta‐analysis. Eur Heart J. 2010;31(17):2156‐2169. 20601393 10.1093/eurheartj/ehq204

[28] Assessment of the Safety and Efficacy of a New Treatment Strategy with Percutaneous Coronary Intervention (ASSENT‐4 PCI) investigators . Primary versus tenecteplase‐facilitated percutaneous coronary intervention in patients with ST‐segment elevation acute myocardial infarction (ASSENT‐4 PCI): randomised trial. Lancet. 2006;367(9510):569‐578. 16488800 10.1016/S0140-6736(06)68147-6

[29] Ellis SG , Tendera M , de Belder MA , et al. Facilitated PCI in patients with ST‐elevation myocardial infarction. N Engl J Med. 2008;358(21):2205‐2217. 18499565 10.1056/NEJMoa0706816

[30] Scheller B , Hennen B , Hammer B , et al. Beneficial effects of immediate stenting after thrombolysis in acute myocardial infarction. J Am Coll Cardiol. 2003;42(4):634‐641. 12932593 10.1016/s0735-1097(03)00763-0

