# Supplementary material for: Rationale and design of the OPTIMAL‐REPERFUSION trial: A prospective randomized multi‐center clinical trial comparing different fibrinolysis‐transfer percutaneous coronary intervention strategies in acute ST‐segment elevation myocardial infarction
Source: Clin Cardiol. 2021 Feb 25;44(4):455–62. doi: 10.1002/clc.23582 (PMC8027583; doi:10.1002/clc.23582)
Supplement: Supplementary file 1 — Appendix S1: Supporting Information [file CLC-44-455-s001.docx]

**Rationale and design of the OPTIMAL-REPERFUSION trial: A prospective randomized multi-center clinical trial comparing different fibrinolysis-transfer percutaneous coronary intervention strategies in acute ST-segment elevation myocardial infarction**

**Hypothesis and objectives**

The primary hypothesis is that STEMI patients with anticipated PPCI delay receiving reduced-dose facilitated PCI strategy will achieve lower composite rate of death, reinfarction, refractory ischemia, congestive heart failure, and cardiogenic shock in comparison to the pharmacoinvasive approach at 30 days. Secondary hypotheses are that reduced-dose facilitated PCI strategy will achieve lower 1-year rate of major cardiovascular adverse events, smaller infarct size, and not increase bleeding risk. The primary objective is to determine whether reduced-dose facilitated PCI strategy is superior to pharmacoinvasive approach in terms of primary endpoints in patients with STEMI and anticipated PPCI delay. The secondary objective is to determine whether reduced-dose facilitated PCI strategy is superior to pharmacoinvasive approach in terms of secondary endpoints in patients with STEMI and anticipated PPCI delay.

**Ethics**

The Ethics Committee of West China Hospital of Sichuan University (Sichuan, China) has approved the protocol. The protocol will also be approved by institutional review boards in other participating centers before recruitment. Any amendments will be submitted and approved by each institutional ethics committee.

**Consent**

Written informed consent to enter the trial and be randomized will be obtained from participants or, in the case of those lacking capacity to consent, from next of kin with legal responsibility. Consent will be obtained after explanation of the aims, methods, benefits and potential hazards of the trial, and before any trial-specific procedures are performed or any blood is taken for the trial. Once the patient regains capacity to consent, persons enrolled via surrogate consent will be re-consented, with care taken to ensure they understand that they are: (1) free to withdraw from the research study; and (2) if they do withdraw, this will not jeopardize their future care. Patients who withdraw will revert to standard of care at the treatment site (usually direct transfer for PCI). It will be made unambiguously clear that the participant (or guardian) is free to refuse to participate in all or any aspect of the research trial, at any time and for any reason, without incurring any penalty or affecting their access to standard treatment available at the recruiting site. Original signed consent forms will be kept by the investigator and documented in the electronic case report form (eCRF), a copy given to the participant or family and a copy placed in the participant’s medical notes.

**Centers**

This study will be conducted in approximately 30 centers. Each center will consist of one PCI-capable center and several PCI-incapable primary hospitals with well-established fibrinolysis-transfer links with pre-defined PCI center. Enrollment will be performed in non-PCI centers. Each participating PCI-capable center will be required to have experience in routine primary angioplasty for myocardial infarction (with past experience of at least 500 PCI procedures per year in the last 3 years and the main operator is qualified for interventional therapy and has independently completed at least 200 PCI cases per year) and to have a 24-h on-call angioplasty team available. The eligible non-PCI centers still need to meet the following criteria: 1) possessing a high-qualified clinical decision support team; 2) with 120- to 180-minutes transfer time to interventional center; 3) having the ability to complete enrollment, informed consent, randomization, and to initiate thrombolytic therapy within 30 minutes after FMC; 4) with a high degree of cooperation and achieving well quality control after research-related training.

**Data collection and monitoring**

Data will be collected at baseline from enrolled patients including demographics, medical history, previous cardiac investigations, and current medication. The time-steps from symptom onset to reperfusion will be accurately recorded. Eighteen-lead electrocardiograms will be obtained at the time points of pre-fibrinolysis, 60-90 minutes after fibrinolysis, early after PCI directly after transfer to the intensive care unit, and after any clinical event thought to represent myocardial infarction and before/after any subsequent revascularization procedure. In addition to routine blood tests, markers of myocardial injury will be measured before thrombolysis, and every 6 hours over 48 hours after system onset. Patients will undergo transthoracic echocardiography before discharge, at 1 month and 12 months. Prospective monitoring of clinical and adverse events starts at randomization and will continue until 12 months.

Security of electronic records will be maintained according to Good Clinical Practice. eCRF data collected and validated using the electronic data capture system will be stored in an electronic database that is protected using a scheme of authentication and encryption, which render subject identity and personal health information unusable, unreadable or indecipherable to unauthorized individuals.

All endpoints and other serious adverse events will be recorded in the eCRF and reported to the coordinating center within 24 hours of first identification. On receipt of notification of any trial adverse or clinical event, the coordinating center will request additional details, specific to the nature of the event. These episodes will be carefully monitored by the trial coordinator and will form part of the information provided at regular intervals to the Clinical Events and Data Monitoring Committees. The ethics committee and institutional clinical risk management team will also be notified accordingly. Clinical endpoints will be adjudicated by a clinical events committee blinded to treatment group assignment.

**Sample size re-estimation**

One interim sample size re-estimation will be conducted when approximately 50% of participants have been recruited to verify if the trial is not under-powered based on potential differences between the observed event and dropout rate pooled across the two arms (i.e. blinded) and the estimates of these pooled rates used in the sample size calculations. To maintain Type 1 error and trial integrity and safeguard the power of the trial, this will be conducted in a blinded way: i.e. the effect size will not be taken into consideration as no details by arm will be provided to the statistician conducting the sample size re-estimation.

**Study committees**

**Executive Committee**

The Executive Committee is composed of Yong He (Chairman), Duolao Wang (Senior statistician, UK), Jianhong Tao, Lin Cai, Jianxiong Liu, Zhongxiu Chen. It provides scientific direction of the study and assesses the study progress. The Executive Committee will meet periodically and be assisted by the Steering Committee. The Executive Committee Chairman is responsible for communicating with the DSMB and sponsor when appropriate.

**Steering Committee**

The Steering Committee is composed of the Executive Committee and investigators from all participating centers, usually the principal investigators of every center. The Steering Committee will meet periodically to assess the progress, provide scientific input and address policy issues and operational aspects of the protocol. Representatives of the sponsor may attend these meetings as non-voting members.

**Data and Safety Monitoring Board**

An independent Data and Safety Monitoring Board (DSMB) will monitor the accruing safety and outcome data regularly. The DSMB will be composed of Qing Zhang (independent cardiologist), Yan Li (independent interventionist), and Guanjian Liu (independent biostatistician). The DSMB operations will be formally separated from the sponsor, the investigators and the Steering/Executive Committee. The DSMB will advise the Chairman of Executive Committee by giving recommendations on trial continuation/discontinuation or aspects of study conduct.

**Clinical Events Committee**

A blinded and independent Clinical Events Committee (CEC) will adjudicate the clinical endpoints. The CEC is composed of three independent interventional cardiologists, Pengcheng He, Chunjian Li, and Kai Xu. The membership will meet periodically and is responsible for reviewing each event as defined in the protocol. Their work is independent and impartial. Such type of review is to ensure accurate counting, assessing, rating, etc. of protocol-defined events.

**Supplemental Table 1. Participating centers and the principal investigators at each center.**

| Participating centers | Principal investigators | Cities in China |
| --- | --- | --- |
| West China Hospital of Sichuan University | Yong He, Zhongxiu Chen | Chengdu, Sichuan |
| Sichuan Provincial People's Hospital | Jianhong Tao | Chengdu, Sichuan |
| Chengdu First People's Hospital | Jun Zhang | Chengdu, Sichuan |
| Chengdu Second People's Hospital | Jianxiong Liu | Chengdu, Sichuan |
| Chengdu Third People's Hospital | Lin Cai | Chengdu, Sichuan |
| Chengdu Fifth People's Hospital | Mingjian Lang | Chengdu, Sichuan |
| Mianyang Central Hospital | Caidong Luo | Mianyang, Sichuan |
| Sichuan Mianyang 404 Hospital | Decai Li | Mianyang, Sichuan |
| The First People's Hospital of Yibin | Chengling Li | Yibin, Sichuan |
| People's Hospital of Leshan | Hongbo Liu | Leshan, Sichuan |
| Leshan Armed Police Corps Hospital | Jianbin Mao | Leshan, Sichuan |
| Xichang People's Hospital | Qiancai Xiang | Xichang, Sichuan |
| The Affiliated Hospital of Southwest Medical University | Zhongcai Fan | Luzhou, Sichuan |
| Affiliated Hospital of North Sichuan Medical College | Zhan Lv | Nanchong, Sichuan |
| Nanchong Central Hospital | Jianping Deng | Nanchong, Sichuan |
| Suining Central Hospital | Xuejun Deng | Suining, Sichuan |
| People's Hospital of Deyang City | Xiaojian Deng | Deyang, Sichuan |
| Zigong First People's Hospital | Wenzhai Cao | Zigong, Sichuan |
| Zigong Forth People's Hospital | Yong Yi | Zigong, Sichuan |
| Ya'an People's Hospital | Haibo Zhang | Yaan, Sichuan |
| Dazhou Central Hospital | Yong Guo | Dazhou, Sichuan |
| The First Affiliated Hospital of Chongqing Medical University | Kanghua Ma | Chongqing |
| Xinqiao Hospital, Army Medical University | Xiaohui Zhao | Chongqing |
| Chongqing Emergency Medical Center | Jun Xiao | Chongqing |
| The Affiliated Hospital of Guizhou Medical University | Wei Li | Guiyang, Guizhou |
| Affiliated Hospital of Zunyi Medical University | Bei Shi | Zunyi, Guizhou |
| The First People's Hospital of Zunyi (the Third Affiliated Hospital of Zunyi Medical University) | Qianfeng Jiang | Zunyi, Guizhou |
| First Affiliated Hospital of Kunming Medical University | Zhaohui Meng | Kunming, Yunnan |
| The Second People's Hospital of Yunnan Province | Xinjin Zhang | Kunming, Yunnan |
| Yan'an Hospital Affiliated to Kunming Medical University | Xuefeng Guang | Kunming, Yunnan |

**Supplemental Table 2. Exclusion criteria for the trial.**

| **Exclusion criteria** |
| --- |
| **1. Fibrinolysis contradictions** |
| Definite hemorrhagic stroke history or stroke of unknown origin at anytime； |
| Any history of central nervous system damage (i.e. neoplasm, aneurysm, arteriovenous malformation, intracranial or spinal surgery); |
| Ischemic stroke in nearly 6 months; |
| Recent trauma to the head or cranium (i.e. < 3 months); |
| Active bleeding or known bleeding disorder/diathesis; |
| Current use of oral anticoagulation (warfarin or new oral anticoagulant); |
| Arterial aneurysm, arterial/venous malformation and aorta dissection; |
| Uncontrolled hypertension, defined as a single BP measurement ≥ 180/110 mmHg (systolic BP ≥ 180 mmHg and/or diastolic BP ≥ 110 mmHg) prior to randomization; |
| Major surgery, biopsy of a parenchymal organ, noncompressible vascular puncture, or significant trauma within the past 2 months (this includes any trauma associated with the current myocardial infarction); prolonged or traumatic cardiopulmonary resuscitation (> 10 minutes) within the past 2 weeks; major surgery pending in the following 30 days; |
| **2. Complex cardiac complication** |
| Evidence of cardiac rupture; |
| Pre-existing heart failure and previous New York heart function classification III-IV； |
| Cardiogenic shock (systolic BP <90 mmHg after fluid infusion or systolic BP<100 mmHg after vasoactive drugs); |
| PCI within previous 6 months or previous bypass surgery; |
| Myocardial infarction in the past year or previously known coronary anatomy not suitable for revascularization; |
| Known acute pericarditis and/or subacute bacterial endocarditis; |
| Hospitalization for cardiac reason within past 48 hours; |
| **3. Severe comorbidity** |
| Other diseases with life expectancy ≤12 months; |
| Any history of severe renal or hepatic dysfunction (hepatic failure, cirrhosis, portal hypertension or active hepatitis); neutropenia, thrombocytopenia; |
| Severe COPD with hypoxemia; |
| **4. Not suitable for clinical trial** |
| Pregnant or lactating; |
| Body weight <40 kg; |
| Known hypersensitivity to any drug that may be used in the study; |
| Inclusion in another clinical trial; |
| Previous enrollment in this study or treatment with an investigational drug or device under another study protocol in the past 7 days; |
| Inability to follow the protocol and comply with follow-up requirements or any other reason the investigator feels would place the patient at increased risk. |

Abbreviations: BP, blood pressure; COPD, chronic obstructive pulmonary disease; PCI, percutaneous coronary intervention.
